# Steady hydrodynamic interaction between human swimmers

**DOI:** 10.1098/rsif.2018.0768

**Published:** 2019-01-23

**Authors:** Zhi-Ming Yuan, Mingxin Li, Chun-Yan Ji, Liang Li, Laibing Jia, Atilla Incecik

**Affiliations:** 1 School of Naval Architecture and Ocean Engineering, Jiangsu University of Science and Technology, Zhenjiang, Jiangsu 212003, People's Republic of China; 2 Department of Naval Architecture, Ocean and Marine Engineering, University of Strathclyde, Glasgow G4 0LZ, UK; 3 School of Marine Science and Technology, Northwestern Polytechnical University, Xi'an, Shaanxi 710072, People's Republic of China

**Keywords:** hydrodynamic interaction, drafting, competitive swimming, wave drag, swimming configuration

## Abstract

This study focuses on the hydrodynamic interaction between two or three human swimmers in competitive swimming. Although the swimming performance of a single swimmer has been widely examined, studies on the interaction between multiple competitive swimmers are very rare. Experiments showed evidence that the drag of a swimmer could be modified by the existence of the other adjacent competitors (Chatard & Wilson. 2003 *Med. Sci. Sports Exerc*. **35**, 1176–1181. (doi:10.1249/01.MSS.0000074564.06106.1F)). The following questions arise: (1) what mechanism determines the interaction; (2) which position experiences drag reduction or drag increase; (3) how much can drag be reduced or increased in a formation? According to the authors' knowledge, such questions have not been addressed by any published literature. Therefore, the main purpose of this study is to find the mechanism of the hydrodynamic interaction between human swimmers and to quantify this interactive effect by using a steady potential flow solver. The free-surface effect was fully taken into account in our calculations. We firstly calculated the wave drag of a swimmer swimming solely in an open swimming pool. Then we calculated the wave drag of the same swimmer when he/she swam in the wake region of one or two leading swimmers. The results showed that the hydrodynamic interaction made a significant contribution to the drafter's wave drag. By following a leading swimmer, a drafter at wave-riding positions could save up to 63% of their wave drag at speed of 2.0 m s^−1^ and lateral separation of 2.0 m. Particularly, when a drafter is following two side-by-side leaders, the drag reduction could even be doubled. To the authors' knowledge, this study is the first to demonstrate that the hydrodynamic interaction between human swimmers can best be described and explained in terms of wave interference effect on the free water surface. When the wave cancellation effect is observed, the wave drag of a drafter could be minimized, and this wave cancellation effect can be achieved only when the drafter is in a wave-riding position.

## 1. Introduction

Pioneering studies have provided fundamental insight into the interactions between a group of animals travelling in formation. Studies on ducklings swimming in formation [1,2], fish in schools [3] and birds flying in a ‘V’ formation or single-file line [4–8] have found the energy consumption of individuals during group locomotion could be reduced. The ‘aid’ that the animal acquired from its companions varies by species. For schooling fish and flying birds, the downwash wake produced by a leader may be used by its followers as a propelling aid [7–13]. But for the ducklings swimming in formation on the free water surface, they benefit from using the waves generated by the mother duck. Inspired by the behaviour of animals in formation, human runners and cyclists use pace lines as the most important race tactic. By travelling in a group, racing cyclists can increase their speed about 0.9–1.8 m s^−1^, while runners can improve about 0.1 m s^−1^ [14–17]. These studies shed light on the performance of human competitive swimmers. Can the following swimmers (referred to as the ‘drafter’ hereafter) benefit from the wakes of the leading swimmers (referred to as the ‘leader’ hereafter), thus reducing the drag and conserving the energy cost? Here, we study the hydrodynamic interaction between two and three swimmers swimming at the same speed and explain the interaction in terms of the wave interference phenomenon.

For a single competitive swimmer, the drag (resistance) is considered to be one of the most important factors which determines his/her swimming performance. In most of competitive swimming styles (apart from butterfly stroke), the total drag *R*_T_ of a swimmer is mainly made up of three components: wave drag *R*_w_ due to wave-making, and skin-friction drag *R*_f_ due to fluid viscosity and pressure drag *R*_p_ arising as a result of distortion of flow outside of the boundary layer [18]. Of course, the spray could also induce a drag. In competitive swimming, the success or failure is usually measured in seconds (long course) or even in hundredths seconds (short course). Therefore, reducing the drag would improve performance. Most of the studies on drag reduction focus on swimmer's body position [19–21], morphology [22,23], swimming technique [24–28] and swimwear technology [21,29–33]. Particularly, the skin-friction drag can be reduced by 2–10% according to Toussaint *et al*. [30] and Koeltzsch *et al*. [34]. Considering the contribution of the skin-friction drag component to the total drag is up to 5% given the high Reynolds numbers (greater than 105) that occur during swimming [35,36], the drag reduced by wearing fast-skin suits is non-significant. The contribution of the other two drag components depends highly on gliding depth. Lyttle *et al*. [37,38] found that there was no significant wave drag when a swimmer was gliding at least 0.6 m underwater. However, the wave drag increases quickly as the swimmer swims closer to the free water surface. It contributes around 50–60% to total drag force in elite swimmers when swimming at the surface [39]. It indicates that if we are able to minimize the wave drag, the total drag can be reduced significantly and the performance of the swimmers can be improved consequently.

**Figure 1.**
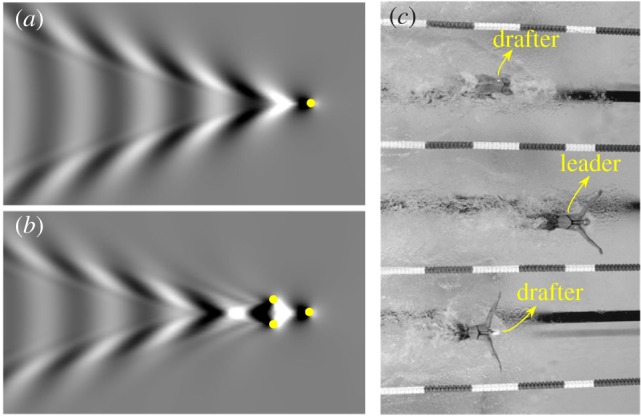
(*a*) The wave pattern generated by a single source point submerged at $H=0.3U^{2}/g$; (*b*) destructive wave pattern generated by three source points submerged at $H=0.3U^{2}/g$ in a V-shape configuration; (*c*) the formation of three swimmers in competitive swimming (https://accidentalokie.files.wordpress.com/2012/07/11239827-essay.jpg). (Online version in colour.)

The wave drag is associated with the waves generated by an advancing swimmer. To reduce the wave amplitude, one effective way is to improve the swimmer's technique. The examples include increasing gliding depth, as mentioned earlier, changing breaststroke technique [25], and optimizing head or finger positions [20,40,41]. Alternatively, a swimmer (drafter) may ‘ride’ the waves generated by his/her adjacent competitors (leaders). By positioning drafter's fore part in a wave trough and aft part in a wave crest, the wave cancellation effect occurs, which will reduce the waves generated by the drafter and minimize the drag. Drag reduction of a drafter has been confirmed by experiments by Chatard & Wilson [42]. The measurements by Janssen *et al*. [43] showed that passive drag and oxygen uptake were significantly reduced when drafting. It has also been confirmed in naval architecture that the wave cancellation effect is beneficial for drag reduction of multihull vessels [44–46]. To demonstrate this wave cancellation effect, we calculated the waves generated by a single translating source point (figure 1*a*), and the waves generated by three source points in an optimal V-shape configuration (figure 1*b*). The transverse waves generated by the two drafters are partly cancelled by travelling in the leader's wake. As a result, the wave energy propagated to the fluid domain is conserved. When this wave cancellation effect occurs among multiple swimmers, the reduced wave energy is equivalent to the energy saved by the drafter. Although the waves generated by a swimmer's three-dimensional body are much more complicated, as shown in figure 1*c*, the wave interference phenomenon can be interpreted by the same principle.

## 2. Methods

In this paper, we are only interested in the wave drag component. No attempt is made here to analyse the other drag components due to the viscosity of the fluid. The main purpose of this paper is to quantify wave drag reduction in formation swimming and find the mechanism of the hydrodynamic interaction between human swimmers. To make the goals achievable, we make the following assumptions:

(1) The skin-friction drag *R*_f_ and pressure drag *R*_p_ of a drafter will be reduced when he/she swims in the low-pressure region created by the leader. This low-pressure region is usually confined within a narrow wake area right behind the leader. As a result, a drag reduction was found in the measurements when a drafter swims in the same lane behind a leader [42]. But in competition pools, the swimmers are swimming side-by-side in different lanes. The lateral separation is sufficiently large to eliminate the effect of wake turbulence. On the other hand, it is well known that the skin-friction drag *R_f_* and pressure drag *R_p_* are mainly determined by three factors: the speed *U*, the area *S_b_* and the shape (or drag coefficient *C_d_*) of the swimmer's immersed body surface. For the same swimmer swimming at the same speed, these three factors can be regarded as the same whether swimming in formation (in different lanes) or alone. Therefore, the difference of total drag in single and formation swimming (in different lanes) is mainly caused by wave-making. This assumption is also adopted by naval architects in catamaran design [44,46].
(2) The passive swimmer, either the drafter or the leader, is assumed to be a rigid and smooth body. The local movement of different parts of the body is not taken into account. The flexibility of an active swimmer's body and the local movement of different body parts will definitely bring changes to the drag, as discussed by Vennell *et al*. [39]. However, this effect is consistent in single and formation swimming. Therefore, only a rigid swimmer model with the arms alongside the body is considered in the present study.
(3) The gliding depth remains constant. Neither sinkage nor trim will be considered in our calculations.
(4) The swimmers are assumed to swim in open water. No attempts are made here to calculate the wave absorbing effect of the lane ropes. Rizk [47] investigated and quantified the efficiency of the wave damping properties of the lane ropes. It was concluded that within the most efficient case of wave damping, the swimming ropes attenuated about 70% wave height transmitted through it. However, at least 30% of the wave energy is still transmitted to the adjacent lanes, which can be used by the drafter as a propelling aid.
(5) Only the primary characteristics of swimmer's body shape are modelled in our calculations. The detailed geometry, e.g. fingers, hands, ears, is not considered in the three-dimensional model.

Based on the above assumptions, the fluid domain can be described by using a velocity potential *φ*. Furthermore, if the water is assumed to be incompressible, it follows that the velocity potential *φ* has to satisfy the Laplace equation:

2.1 $$\frac{\partial^{2}\varphi }{\partial x^{2}}+\frac{\partial^{2}\varphi }{\partial y^{2}}+\frac{\partial^{2}\varphi }{\partial z^{2}}=0.$$

**Figure 2.**
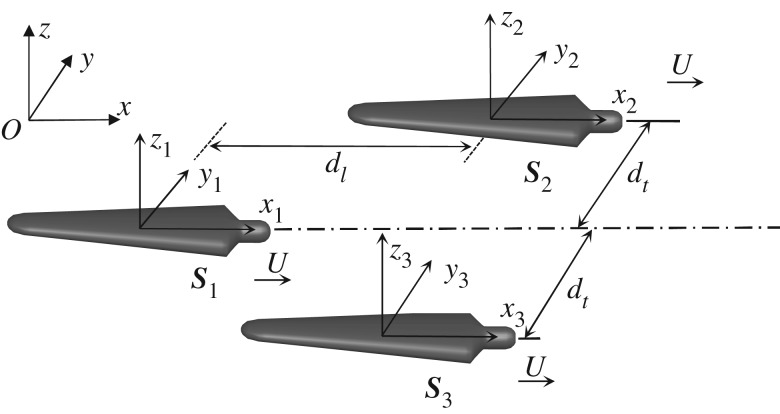
Coordinate systems.

A three-dimensional potential flow theory, which is widely used in ship hydrodynamics, can be used in the present study to calculate the wave drag of a swimmer. It should be noted that the drafter ***S***_1_ and leader ***S***_2_, ***S***_3_ are assumed to swim at the same speed U and same direction in formation swimming. Thus, the overtaking or encountering situation will not occur. Two kinds of reference systems are established with the global earth-fixed *O*-*xyz* and local body-fixed *o*-*x_i_y_i_z_i_*, (*i* = 1, 2, 3…) references in figure 2. The lateral and longitudinal separation distance between the drafter ***S***_1_ and leader ***S***_2_ are defined as *d_t_* and *d*_l_, respectively. The depth of the water is 2 m, which can be regarded as deep water in the calculations. The velocity potential is time-independent in the moving frame. It implies the hydrodynamic interaction can be treated as a steady problem, as the swimming speed is constant. By combining the dynamic and kinetic free-surface conditions, the time-independent linearized steady free-surface condition [48] can be written as

2.2 $$U^{2}\frac{\partial^{2}\varphi }{\partial x^{2}}+g\frac{\partial \varphi }{\partial z}=0,$$

where *g* is the acceleration due to gravity. The body surface boundary condition follows from the requirement that there be no flow through the body surface. This means

2.3 $$\frac{\partial \varphi }{\partial n}=Un_{1},$$

where $n=(n_{1},n_{2},n_{3})$ is the unit normal vector inward on the wet body surface. Besides, a radiation condition is imposed on the control surface to ensure that waves vanish at upstream infinity

2.4 $$\varphi \to 0,\;\;\zeta \to 0\;\;as\;\sqrt{x^{2}+y^{2}}\to \infty ,$$

where *ζ* is the wave elevation. A Rankine source panel method [49] is used to solve the boundary value problem in equations (2.1)–(2.4). The details of numerical implementation are demonstrated in Yuan *et al*. [50]. The same in-house developed multibody hydrodynamic interaction program MHydro, which has been extensively validated against ship model tests, is deployed in the present study to predict the interactions in a swimming pool. Special care should be taken to implement a suitable open boundary condition to satisfy equation (2.4). In numerical calculations, the computational domain is always truncated at a distance away from the moving body. A second-order upwind difference scheme is applied on the free surface to obtain the spatial derivatives. In this way, the waves could propagate to the far-field without reflection.

Once the unknown potential *φ* is solved, the steady pressure distributed over the ship hull can be obtained from linearized Bernoulli's equation:

2.5 $$p=\rho U\frac{\partial \varphi }{\partial x},$$

where *ρ* is the water density. Integrating the pressure over the hull surface, the forces (or moments) can be obtained by

2.6 $$F_{i}=\int \int_{S}\;pn_{i}\;ds,\;i=1,\;\;2,\ldots ,\;\;6,$$

where

2.7 $$n_{i}=\left\{\;\begin{array}{c} n,\;i=1,\;\;2,\;\;3\\ x\times n,\;i=4,\;\;5,\;\;6. \end{array}\right.$$

The wave drag *R*_w_ is equivalent to the force component in the negative *x*-axis (*i* = 1). The wave elevation on the free surface can be obtained from the dynamic free-surface boundary condition in the form

2.8 $$\zeta =\frac{U}{g}\frac{\partial \varphi }{\partial x}.$$

## 3. Numerical modelling

### 3.1. Validation of the numerical model

**Figure 3.**
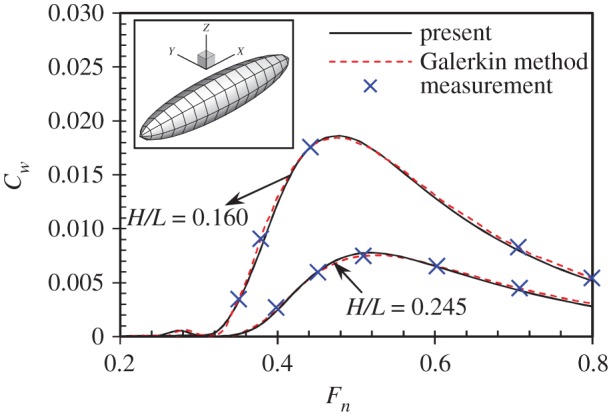
Wave drag of a submerged prolate ellipsoid of diameter-to-length ratio *D*/*L* = 0.2 at different submerged depths. The red dash curves indicate the numerical results calculated by using a Galerkin method to solve the Neumann–Kelvin problem [51]. The blue crosses indicate the experimental results measured by Farell & Guven [52]. The present calculations are shown in black solid curves. (Online version in colour.)

The present methodology and numerical programme is firstly applied to calculate the wave drag of a submerged ellipsoid at different submerged depths. The wave-making resistance of a submerged ellipsoid is a classic hydrodynamic problem, which has been widely studied. The numerical results calculated by Doctors & Beck [51], as well as the experimental results measured by Farell & Guven [52], are used here to validate the present calculations. The comparisons are shown in figure 3. The Froude number *F_n_* ($F_{n}=U/\sqrt{gL}$) is used as the non-dimensional speed. The wave drag is non-dimensionalized by using the following formula:

3.1 $$C_{w}=\frac{R_{w}}{0.5\rho U^{2}S},$$

where *S* is the area of the wet body surface. Two submerged depths are simulated: *H*/*L* = 0.160 and 0.245, where *H* is the submerged depth, *L* is the length of the ellipsoid. The comparisons show very good agreement between the present calculations and measurements, as well as Doctors and Beck's numerical results. It implies the present methodology and numerical programme are applicable to predict the wave drag of a swimmer moving close to the free water surface with various speeds. The results also indicate that the wave drag increases rapidly as the ellipsoid moves closer to the free water surface. It coincides with Lyttle's studies on human swimmers [37,38].

**Figure 4.**
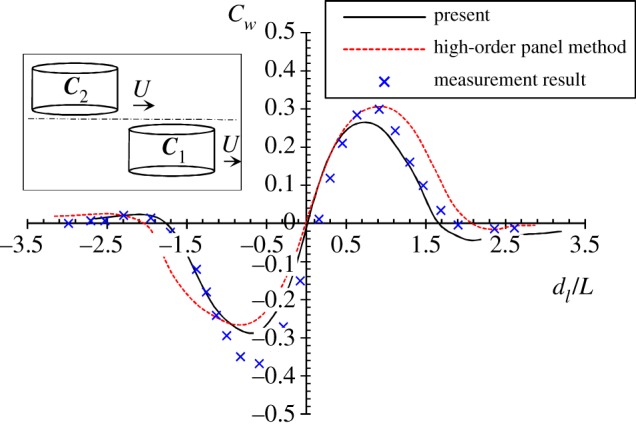
The wave drag on cylindroid ***C***_2_ when it is moving parallelly with ***C***_1_ at *F_n_* = 0.217. The negative *d*_l_ values denote that ***C***_2_ is the drafter. As ***C***_2_ becomes the leader, *d*_l_ becomes positive. The red dash curve indicates the numerical results calculated by using a NURBS-based high-order panel method [54]. The blue crosses indicate the experimental results measured by Oltman [53]. The present calculations are shown in black solid curves. (Online version in colour.)

To validate the capacity of the present methodology and numerical programme in predicting the hydrodynamic interaction between two swimmers, a case study on two identical cylindroids moving in parallel at *F_n_* = 0.217 is conducted. The semi-major axis of the ellipses is *a* = 0.4 m, and the ratio of the semi-major axis to the semi-minor axis is *a*/*b* = 8.0. Water depth is *h* = 3 m, and the draught is 1.47 m. The separation distance is *d_t_* = 5.0*b*. The experimental data measured by Oltman [53] and the numerical results calculated by using a high-order panel method [54] are compared with the present calculations, as shown in figure 4. Generally, the present calculations show good agreement with the measurements, as well as with Xu's numerical results. An interesting finding is that a very large negative wave drag (the force is pointing towards the moving direction) can be observed at *d*_l_/*L* = −0.75. It implies that when an object (drafter) is located in the wake of the other object (leader), the hydrodynamic interaction can be used by the drafter as a propelling aid. Similar findings were also observed in laboratory experiments of two ships travelling side-by-side [50,55]. It should be noted that the hydrodynamic interaction between two cylindroids travelling at low Froude number in this case study is dominated by the near-field waves. In competive swimming, the Froude number of the swimmers is much higher (around 0.4–0.5). As a result, the far-field waves (or Kelvin waves) could be the most important factor that determines the interactive forces. This will be discussed later.

### 3.2. Description of the swimmer model

**Figure 5.**
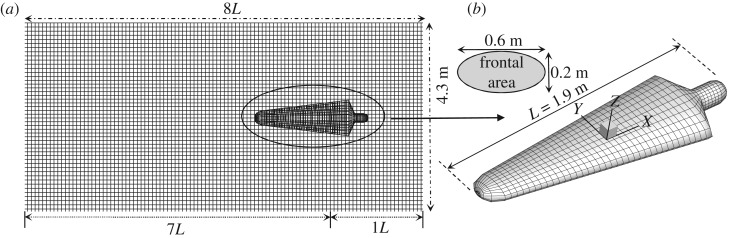
Panel distribution on the computational domain. In single swimmer case, there are 13 717 panels distributed on the entire computational domain: 2141 on the wetted body surface, 11 576 on the free-surface. The free-surface is truncated at 1*L* upstream and 7*L* downstream with regard to the body-fixed frame on the swimmer model. The local coordinate system is fixed on the moving body with its positive *x*-direction pointing towards the head, positive *z*-direction pointing upwards and *z* = 0 on the undisturbed free-surface. (Online version in colour.)

In numerical modelling of animal swimming, the animal's three-dimensional body shape is usually idealized as some simplified geometry. Based on Tuck and Newman's slender body theory [56], Weihs [57] modelled a dolphin as an oblate ellipsoidal shape with an aspect ratio of about 6, in order to investigate the hydrodynamics of dolphin drafting. A similar approach was also used by Lang [58], defining the body shape of a dolphin as an ellipsoid with an added tail region. Compared with the dolphin body, the shape of the swimmer model is much more complex. Westerweel *et al*. [59] conducted measurements of a scaled swimmer model by using a simplified model with the arms alongside the body. A similar simplification is made for the present swimmer model. The three-dimensional numerical swimmer model is shown in figure 5*b*. It should be noted that the total wet body surface of the numerical swimmer model is *S* = 1.65 m^2^ without considering the different swimming movements. It is smaller than the area of a real competitive swimmer (*S* = 1.9 m^2^) when the arms and legs are fully exposed to the water. The computational domain of the numerical model is shown in figure 5*a*. All the boundaries are discretized into a number of quadrilateral panels with constant source density. To capture the far-field waves propagating downstream, the free-surface is truncated at least 7 l behind the swimmer. The water depth of the swimming pool is 2 m.

## 4. Results and discussions

### 4.1. Wave drag of a single swimmer

The total drag of a single swimmer has been extensively investigated both numerically and experimentally. However, only a few studies have been conducted to quantify the wave drag component. The contribution of the wave drag to the total drag varies greatly in these studies. Vorontsov & Rumyantsev [60] suggested that 5% of drag was due to waves at 2 m s^−1^. Toussaint *et al*. [61] found the wave drag amounted to 12% of the total drag. These studies significantly underestimate the wave drag contribution. It was assumed that the wave drag was negligible when the swimming speed was below 1.6 m s^−1^ (*F_n_* < 0.35). However, it is well known in naval architecture that for a surface vessel, the wave drag becomes dominant at *F_n_* > 0.3 [62]. More specifically, the wave drag contributes up to 55% of the total drag at *F_n_* = 0.35 for a surface-piercing body. The contribution increases to more than 70% at *F_n_* = 0.45. It should be noted that the wave drag of a surface-piercing body is larger than that a fully submerged one. Even for a fully submerged body, both experimental measurements and numerical calculations confirm that the wave drag varies a lot at different submerged depths, as shown in figure 3. In order to obtain reliable wave drag results, the submerged depths must be taken into account. Lyttle *et al*. [37] investigated the effect of submerged depth and velocity on drag during the streamlined glide. Their experimental results suggest that at 2.2 m s^–1^, the total drag is 20% lower at 0.6 m depth than at the surface. The measurements by Vennell *et al*. [39] show that the wave drag is 50–60% of the total drag on elite swimmers swimming close to the surface at 1.7 m s^−1^, which is much higher than any previous estimate. All the above-mentioned experimental studies obtain the wave drag indirectly by subtracting the skin and form drag from the total measured drag. The skin and form drag are assumed to be equal to the total drag when the submerged depth is very large [39], or when the swimming speed is below 1.6 m s^−1^ [61]. However, in ship hydrodynamics, it is straightforward to calculate the wave-making resistance (or the wave drag) by a well-established potential flow theory. As the viscosity of the fluid is not considered, the resistance calculated by solving the Laplace equation in equation (2.1) is equivalent to the wave drag. In this study, the same methodology used in naval architecture will be applied to calculate the wave drag on human swimmers.

**Figure 6.**
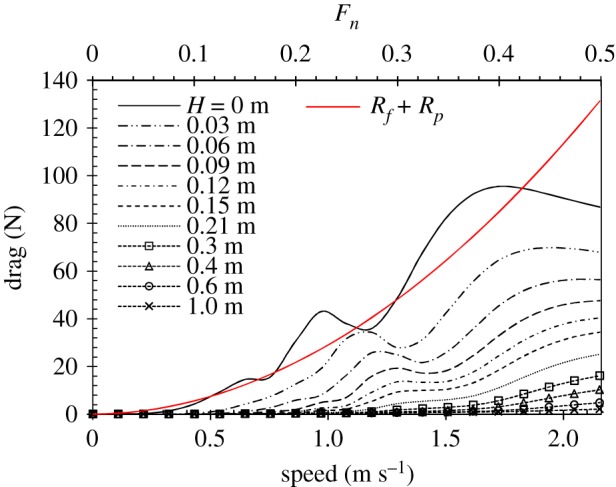
Wave drag of a single human swimmer. The black curves indicate the wave drag at different submerged depths. *H* = 0 indicates that the highest point on the body surface is on the free-surface. The red curve indicates the sum component of the frictional and pressure drag. (Online version in colour.)

The wave drag on a single swimmer is shown in figure 6. When the swimmer is swimming near the free-surface (*H* = 0.0–0.2 m), the wave drag decreases rapidly as the submerged depth increases. The curves exhibit ‘bumps’ and ‘hollows’(which are also called amplification and cancellation effects) due to the interference between bow- and stern-waves [63]. These ‘bumps’ and ‘hollows’ shift to higher velocities and become less distinct as the submerged water depth increases. For a competitive swimmer, the non-dimensional velocity (Froude number) is usually larger than 0.35. Therefore, these ‘bumps’ and ‘hollows’ will not have prominent influence on the swimmer's performance. At moderate submerged depth (*H* = 0.2–0.4 m), the wave drag continues to decrease with a slower rate as the submerged depth increases. The ‘bumps’ and ‘hollows’ phenomenon disappears, and the wave drag is only 10–20% of that at *H* = 0 m. At the submerged depth of 0.4 m or larger, the contribution of the wave drag is very small and it is usually neglected in most of the studies on human swimmers. In some experimental studies [18,39], the contribution of the other two drag components (the skin-friction drag *R*_f_ and pressure drag *R*_p_) is measured by towing the mannequin below the *H* = 0.6 m. The wave drag results of a single swimmer shown in figure 6 are consistent with the experimental measurements, which will be used in the next session to non-dimensionalize the wave drag of the same swimmer when swimming alongside the other swimmers.

To assess the contribution of the wave drag to the total drag, the contribution of the other two drag components, namely the skin-friction drag *R*_f_ and pressure drag *R*_p_, should be quantified. The skin-friction coefficient can be determined by the ITTC 1957 correlation line for turbulent flow [64].

4.1 $$C_{f}=\frac{0.075}{{\left(\log(Re)-2\right)}^{2}},$$

where *Re* = *UL*/*ν* is the Reynolds number of the body, *ν* is the kinematic viscosity of the water. The skin frictional resistance then can be calculated by

4.2 $$R_{f}=0.5\rho U^{2}SC_{f}.$$

The form drag *R_p_* is

4.3 $$R_{p}=0.5\rho U^{2}A_{p}C_{p},$$

where *C*_p_ is the form drag pressure efficient. For an elliptical bluff body, *C*_p_ is defined as 0.3 [65]. *A*_p_ is the projected area in the *y*–*z* plane.

The curve of frictional and pressure drag in figure 6 shows the increased drag with speed. When a swimmer is swimming at the free water surface (*H* = 0 m), the contribution of the wave drag and the other two components are of similar magnitudes at low swimming speed (*U* < 1.3 m s^−1^). At medium speed (1.3–1.8 m s^−1^), the wave drag is the largest drag, contributing up to 50–60% of the total. It coincides with the measurements by Vennell *et al*. [39]. At high swimming speed (*U* > 1.8 m s^−1^), the wave drag experiences a decrease with the speed, while the frictional and pressure drag keeps increasing and gradually dominates the total drag. As the submerged depth increases, the contribution of the wave drag drops rapidly. The results in figure 6 clearly show how the submerged depth changes the wave drag and its contribution to the total drag. In the next section, all the results and discussions are based on submerged depth of *H* = 0 m, when the swimmer is just immersed below the free water surface. No attempts are made to investigate the surface-piercing swimmers.

### 4.2. Hydrodynamic interaction between two swimmers in formation swimming

In the last section, we obtained the wave drag of a swimmer swimming alone in open calm water, which is denoted by *R*_ws_. When the same swimmer swims at a certain position around another swimmer, the wave drag is denoted by *R*_w_. The wave drag reduction coefficient can be expressed as

4.4 $$C_{\mathrm{DR}}=\frac{R_{\mathrm{ws}}-R_{w}}{R_{\mathrm{ws}}}\times 100\text{\%}.$$

The wave drag reduction coefficient *C*_DR_ can be used as an indicator to show the hydrodynamic interactive effect. *C*_DR_ > 0 indicates a reduction of wave drag due to the hydrodynamic interaction; *C*_DR_ < 0 represents an increase in the wave drag of a swimmer caused by the presence of the other swimmer(s). No interaction is expected when *C*_DR_ = 0. When *C*_DR_ > 100%, the wave drag turns to be a thrust force, which is in the same direction of moving.

**Figure 7.**
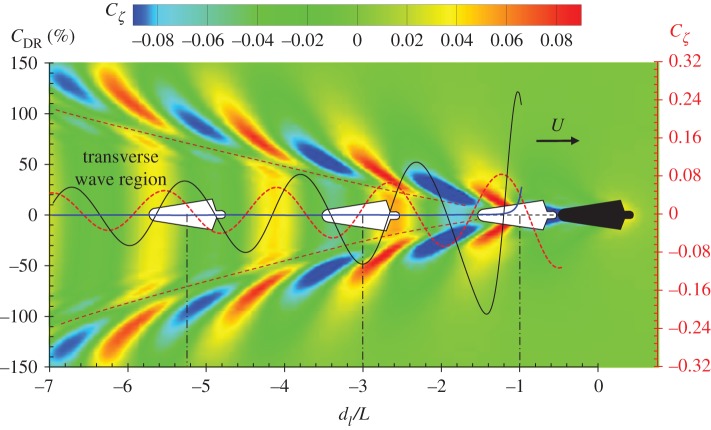
Wave drag reduction coefficient when a drafter swims right behind a leader at *U* = 2.0 m s^−1^. The black solid curve is the *C*_DR_ curve of the drafter, while the blue solid curve is the *C*_DR_ curve of the leader. The colour contour indicates the wave pattern generated by the leader. The red dash curve is the wave profile at the central line behind the leader. The *x*-axis is the non-dimensional distance *d*_l_/*L*. *C_ζ_* is the non-dimensional wave elevation, $C_{\zeta }=\zeta g/2\pi U^{2}$. (Online version in colour.)

First of all, we calculate the wave drag reduction coefficient when a drafter is swimming right behind a leader (the transverse separation *d_t_* = 0) by varying the longitudinal distance *d*_l_. The result of *C*_DR_ is shown in figure 7. The drag reduction curve exhibits distinct fluctuations when the drafter swims towards the leader from −7*L* to −1*L* downstream. The amplitude of the fluctuations becomes larger as the drafter gets closer to the leader. A maximum wave drag reduction of 125% occurs where the drafter's head is almost touching the leader's feet at *d*_l_/*L* = −1.08, indicating the wave drag turns to be a thrust force which pulls the drafter forward. This agrees with the experimental measurements by Chatard & Wilson [42] which concluded that the optimal distance behind the leader was between 0 and 50 cm. In this position, the drafter could significantly save energy by using the waves generated by the leader. It should be noted that swimming at *d*_l_/*L* = −1.08 is very difficult because of the leader's kick rhythm [42]. The kick, and in particular the six-beat kick, can create more bubbles or/and turbulence and induce a visual and arm sweep handicap for the draftee [66]. However, the hydrodynamic interaction does not have a positive effect on the drafter at all positions. When the drafter lags behind the leader slightly at *d*_l_/*L* = −1.45, this interactive effect becomes negative. The wave drag is amplified by the interactive force, which means the drafter has to consume more energy to overcome the extra resistance. It is interesting to find that the trough and crest values of *C*_DR_ appear alternately with a constant interval, fluctuating around *C*_DR_ = 0. This feature of *C*_DR_ curve is very similar to harmonic water waves, which have a constant wavelength. To further investigate the relationship between *C*_DR_ and the free-surface waves, we calculated the Kelvin waves generated by the leader, which are plotted in figure 7 as the background contour. The wave profile at the central line (moving path) of the domain is also shown in the same figure. These results confirm that the interval between the trough and crest of the *C*_DR_ curve is the same as the wavelength of the transverse Kelvin waves, which can be calculated by $2\pi U^{2}/g$. However, these two curves are not in phase. The maximum wave drag reduction is observed when the drafter's fore part is in the wave trough while the aft part is in the wave crest, for example, at *d*_l_/*L* = −1.08, and −5.23. The physical observations of the wave-riding behaviour of dolphins (when chasing boat waves) and ducklings (when following the mother duck) in nature confirm the benefit of this wave-riding configuration [1,67]. Theoretically, it can be explained by the water wave theory. The crest on the free water surface corresponds to a relatively higher pressure under the wave crest profile, while the wave trough corresponds to a lower pressure. According to equation (2.6), the wave drag can be calculated by the pressure integral over the body surface. As the normal vector *n* in the fore part of the drafter is pointing backwards, a lower pressure distribution over the fore part will lead to a smaller backward force (resistance). On the other hand, the normal vector *n* in the aft part is pointing forwards, a higher pressure distribution over the aft part will lead to a larger forward force (propulsion). If the amplitude of the thrust force integrated over the aft part is larger than the resistance integrated over the fore part, a total thrust force can be expected, which is the case shown in figure 7 at *d*_l_/*L* = −1.08. Conversely, if the drafter's fore part is in the wave crest while the aft part is in the wave trough, an extra resistance will be added, which gives rise to the total wave drag, as shown in figure 7 at *d*_l_/*L* = −3.0. The wave amplitude is damped as the waves propagate to the far-field. As a result, the amplitude of the wave drag reduction coefficient reduced as the drafter moves further away from the leader. Another interesting finding is that the wave drag of the leader is also reduced when the drafter gets very close to the leader. A maximum wave drag reduction of 25% is observed at *d*_l_/*L* = −1 when the drafter's head just touches the leader's feet. As the separation increases, this benefit of the leader's drag reduction diminishes rapidly. At *d*_l_/*L* < −1.25 (or at the separation larger than 0.5 m), the leader could hardly experience any wave drag reduction. It can also be explained by the water wave theory. For a body (either ship or human swimmer) moving close to the free water surface, the pressure in the bow (or head) is usually high. As a result, a wave crest is always observed in the bow area. When the drafter approaches the leader, the wave crest accompanied by the drafter's head will modify the pressure distribution over the leader's aft part, creating a wave-riding configuration for the leader, hence pushing the leader forward. The wave crest in front of the bow (or head) vanishes quickly upstream. When the separation between the drafter's head and the leader's feet is larger than 0.5 m, the drafter's bow wave will not have any influence on the leader's wave drag. A similar phenomenon was also confirmed by experimental measurements [59].

**Figure 8.**
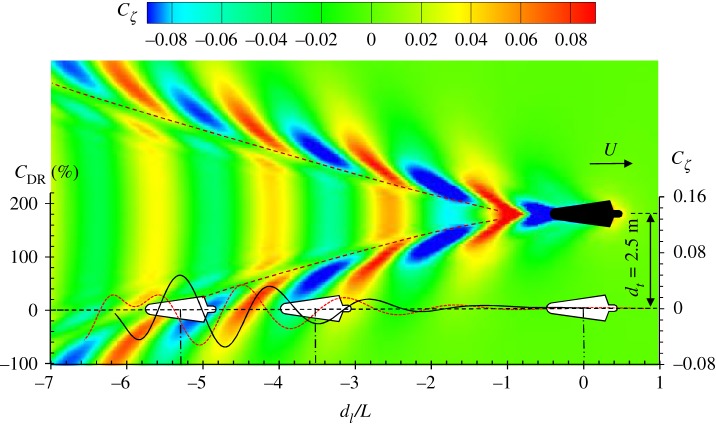
Wave drag reduction coefficient (black solid curve) of a drafter when he/she swims alongside a leader at *U* = 2.0 m s^−1^. The colour contour indicates the wave pattern generated by the leader. The red dash curve is the wave profile at the moving path of the drafter. (Online version in colour.)

When the drafter is swimming right behind the leader, the hydrodynamic interactive force is mainly induced by the transverse wave component generated by the leader. The results in figure 7 explain how these transverse waves influence the wave drag of a drafter. But in competitive swimming, each swimmer must stay in his/her lane, swimming in parallel with a certain transverse distance *d_t_*. As shown in figure 8 at *d_t_* = 2.5 m, when the position of the drafter changes from −7*L* to −1*L*, the drafter has to pass through the transverse waves, the divergent waves and eventually reach a non-disturbed region. Therefore, the hydrodynamic interaction is more complicated. Figure 8 shows the result of *C*_DR_, where the lateral separation between the drafter and leader is *d_t_* = 2.5 m. By varying the longitudinal position, the *C*_DR_ curve exhibits fluctuations around *C*_DR_ = 0. The most violent fluctuations can be observed at −6 < *d*_l_/*L* < −4. This corresponds a region covered by the leader's divergent waves. A maximum wave drag reduction of 64% can be found at *d*_l_/*L* = −5.3 where the drafter's fore part is in the wave trough while the aft part in wave crest. From the results shown in figure 6, it is found that the wave drag comprises about 43% of the total drag at *U* = 2.0 m s^−1^. Then it can be estimated that the drafter can save up to 28% of the total drag if he/she is located in the wave-riding position after a leader. Of course, this estimation is based on the open water assumption, where the lane ropes are not considered. The wave drag increases by 78% if the drafter swims at *d*_l_/*L* = −4.7 due to undesired interaction. The amplitude of the *C*_DR_ curve is not as large as that shown in figure 7, indicating the hydrodynamic interaction induced by leader's transverse waves is more prominent than that induced by the divergent waves. The interactive force gradually vanishes after the drafter is completely out of the Kelvin wake. At *d*_l_/*L* > −1, the hydrodynamic interaction can be negligible. When the drafter and leader are swimming side-by-side (*d*_l_/*L* = 0), no hydrodynamic interaction is observed. The results shown in figure 8 confirm the importance of position in formation swimming. In competitive swimming, the drafter is supposed to be able to sense the drag difference and reposition him/herself to a drag-reduced region to preserve energy during competition.

**Figure 9.**
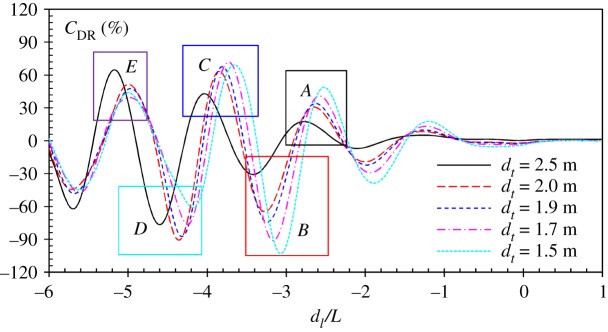
Wave drag reduction coefficient of a drafter when he/she swims alongside a leader at different transverse distances at *U* = 2.0 m s^−1^. The *x*-axis is the non-dimensional longitudinal distance. (Online version in colour.)

The lanes of the World Championship pools are usually 2.5 m wide. If the adjacent swimmers maintain their courses at the mid-lane, the lateral separation between them is 2.5 m; this is the case we showed in figure 8. However, it is commonly observed in competitive swimming that the swimmers may not be able to keep their course at the mid-lane. In order to find how the transverse separation alters the hydrodynamic interaction, we calculate the wave drag reduction coefficient of a drafter at various *d_t_*. The results are shown in figure 9. Similar to the result of *d_t_* = 2.5 m shown in figure 8, the *C*_DR_ curve exhibits fluctuations around *C*_DR_ = 0. The most significant disturbance occurs when the drafter swims at the leader's divergent wave region. At different lateral separations, the drafter encounters the leader's divergent wave at different longitudinal positions. From the colour contour shown in figure 8, it can be seen that as *d_t_* increases, the longitudinal position of entering the divergent wave region is shifted towards larger *d*_l_/*L*. As a result, a phase shift of *C*_DR_ curves can be observed at different *d_t_*.

At small lateral separation, for example *d_t_* = 1.5–1.7 m, a minimum *C*_DR_ of −103% is found in region *B*, while the maximum *C*_DR_ is found in region *C*. When the lateral separation increases, for example, *d_t_* > 1.9 m, the maximum *C*_DR_ remains in region *C*, while the minimum value shifts from region *B* to region *D*. In a swimming competition, the most interesting position is in region *C* or *E*, where the drafter can experience maximum wave drag reduction. In region *C*, the peak value of *C*_DR_ curves varies from 60% to 70%. The discrepancy between the peak values at different *d_t_* is not very obvious in region *C*, indicating that the wave drag reduction is not strongly sensitive to the lateral separation.

**Figure 10.**
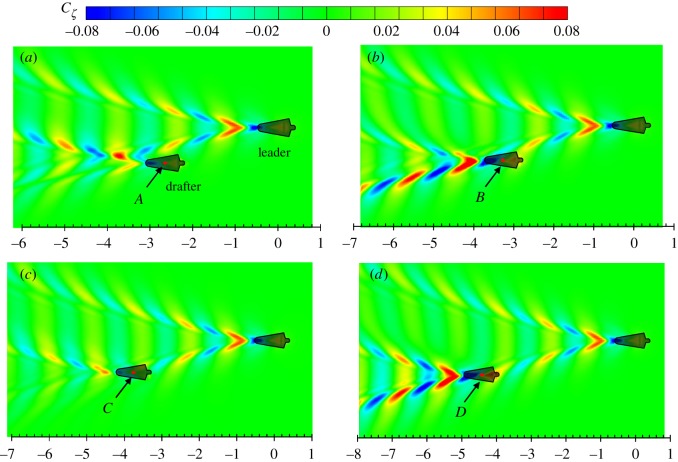
Wave patterns generated by two swimmers at *d_t_* = 2.0 m and *U* = 2.0 m s^−1^. The *x*-axis is the non-dimensional longitudinal distance *d*_l_/*L*. Four typical positions are selected, namely *A*, *B*, *C* and *D*, which represent the peak values in corresponding boxed regions in figure 9. (Online version in colour.)

The results in figures 7 and 8 show that when a drafter is located in the wave-riding position, the wave drag reduction coefficient reaches the maximum value. It can be explained by the pressure integral based on the potential flow theory, which has been explained previously. Here, attempts are made to explain this drag-reducing and drag-increasing phenomenon from another perspective: wave interference. The work done by a swimmer to overcome the wave drag can be transferred into the energy of the Kelvin waves on the free water surface, which is proportional to *ζ*^2^. For a swimmer swimming alone in unrestricted water, *ζ* is mainly determined by swimmer's body shape, posture, speed and submerged depth. The relative position becomes another factor which affects the free-surface elevation if two or more swimmers are swimming in close proximity. The results in figure 10 clearly show how the wave patterns are affected by the drafter's position. Four typical positions are selected, namely *A*, *B*, *C* and *D*, which represent the peak values in corresponding boxed regions of figure 9. In positions *A* and *C*, the drafter takes advantage of wave-riding position to achieve maximum wave drag reduction. In these two positions, a destructive wave interference phenomenon can be observed, where the waves generated by the swimmers are 180° out of phase. The starboard divergent waves of the leader are partly cancelled by the drafter's starboard divergent waves. This effect can be referred to as partial divergent wave cancellation. As a result, the free-surface elevation in the starboard wake of the drafter is reduced, hence conserving energy. This wave cancellation effect has been proved to have a beneficial effect on multihull configuration in order to minimize the wave resistance of a multihull vessel [46,68]. Conversely, if the drafter is located in positions *B* and *D*, the starboard divergent waves generated by the swimmers are in phase. More energy is dissipated in terms of the amplified waves, which requires the drafter to do extra work in order to overcome the increased wave drag. Obviously, positions *B* and *D* are the most undesirable positions in formation swimming. To ‘escape’ from these drag-increased positions, the drafter has to generate an additional thrust to move towards positions *A* and *C* where the wave drag can be minimized.

The portside divergent waves generated by the two swimmers propagate in parallel to the far-field, and they never overlap. However, the portside divergent waves generated by the drafter could also interact with the transverse waves generated by the leader. These two wave systems have different properties in terms of propagation direction and wave length. As a result of superposition, the portside divergent waves of the drafter may be amplified (figure 10*a,d*) or cancelled (figure 10*b,c*). For the high-speed moving body (*F_n_* > 0.4), the divergent wave energy is much higher than the transverse wave energy. Thus, the magnitude of the divergent-transverse wave interference is less than the divergent–divergent wave interference.

### 4.3. Formation swimming of three swimmers

**Figure 11.**
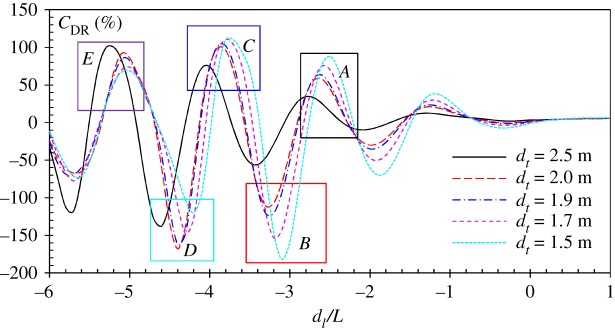
Wave drag reduction coefficient of a drafter swimming in the wake of two side-by-side leaders at both sides at *U* = 2.0 m s^−1^. Different curves correspond to various transverse distances. The *x*-axis is the non-dimensional longitudinal distance between the leaders and the drafter. (Online version in colour.)

In a swimming competition, the hydrodynamic interaction does not only occur between two swimmers. Apart from the swimmers at the first and last lanes, a swimmer usually interacts with the other two adjacent swimmers. The hydrodynamic interaction between three swimmers is very interesting. There are various possible configurations of three swimmers in a formation, among which the V-shape configuration is of particular interest. As shown in figure 2, when a drafter is located in the wake of two leaders at both sides, he/she may achieve more wave drag reduction by using the waves produced by two leaders. The results of *C*_DR_ in a V-shape configuration are shown in figure 11. Similar fluctuations of *C*_DR_ curves are observed in V-shape formation swimming. Compared with the two-swimmer case (figure 9), the amplitudes of the *C*_DR_ curves shown in figure 11 are much higher. For example, at *d_t_* = 2.0 m, the maximum and minimum wave drag reduction are 102% and −167%, respectively in the three-swimmer case, while in the two-swimmer case, the maximum and minimum values are 64% and −90%. The corresponding longitudinal separations in the three-swimmer and two-swimmer cases are consistent. The most interesting position is also found in region C or E, where the drafter can experience a maximum wave drag reduction of up to 110%. As indicated in equation (4.4), when the wave drag reduction is larger than 100%, the wave drag turns to be a thrust force, which pushes the drafter forward. The results in figure 11 indicate the drafter could potentially save more energy by following two side-by-side leaders. From the results shown in figure 6, it is found that the wave drag comprises about 43% of the total drag at *U* = 2.0 m s^−1^. Then it can be estimated that in open water races, the drafter can save up to 50% of the total drag if he/she is swimming in the right position in a V-shape configuration.

**Figure 12.**
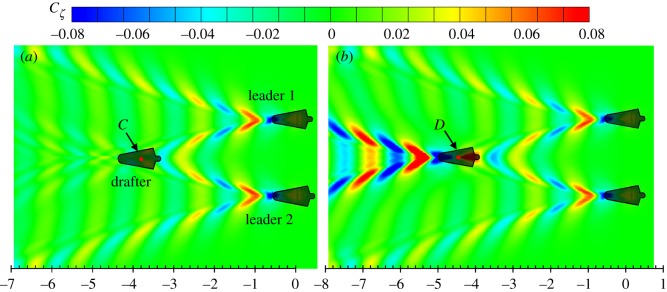
Wave patterns generated by three swimmers in a V-shape configuration at *d_t_* = 2.0 m and *U* = 2.0 m s^−1^. (*a*) Drafter is located at position *C*; (*b*) drafter is located at position *D*. The *x*-axis is the non-dimensional longitudinal distance *d*_l_/*L*. (Online version in colour.)

The results in figure 11 show that the drafter can save 102% of wave drag at *d*_l_/*L* = −3.8 (position *C*). The wave drag increases 167% when the drafter is located at *d*_l_/*L* = −4.4 (position *D*). As discussed before, the wave drag reduction and wave drag increase can be explained by the wave interference phenomenon on the free water surface. Figure 12 compares the wave patterns generated by three swimmers in a V-shape configuration when the drafter is located at *C* and *D*, respectively. Destructive wave phenomenon can be observed in figure 12*a* when the drafter takes the wave-riding position. With the head and shoulders located in the troughs of the divergent waves generated by the leaders, the drafter generates a divergent wave system which is 180° out of phase with Leader 1's starboard divergent waves and Leader 2's portside divergent waves. As a result of superposition, the divergent wave system behind the drafter can hardly be observed. This effect can be referred to as full divergent wave cancellation. Compared with the partial divergent wave cancellation effect in two-swimmer formation swimming, it is obvious that the full divergent wave cancellation could achieve a higher wave drag reduction (almost twice), hence saving more of the drafter's energy. On the contrary, if the drafter is located in position *D*, the divergent waves generated by the three swimmers are in phase. The amplified waves will dissipate more energy, which requires the drafter to do more work in order to overcome the increased wave drag. The results in figures 11 and 12 confirm that the interaction between three swimmers could be more significant than that between two swimmers.

**Figure 13.**
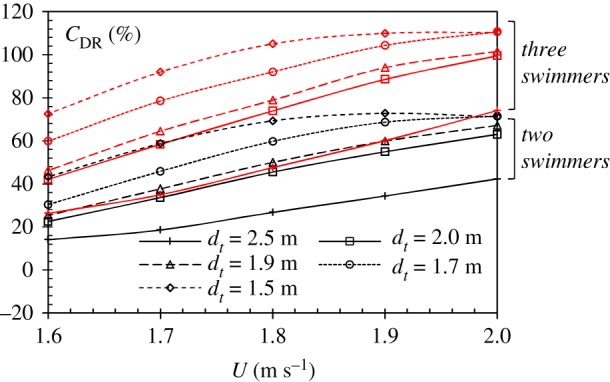
Maximum wave drag reduction coefficient of a drafter in formation swimming. The black curves indicate the maximum *C*_DR_ of a drafter swimming in region *C* in a two-swimmer configuration; the red curves indicate the maximum *C*_DR_ of a drafter swimming in region *C* in a three-swimmer configuration. (Online version in colour.)

The results in figure 13 show the maximum wave drag reduction in formation swimming, varying with speed *U* and transverse separation *d_t_*. The two groups of curves (red and black), representing two and three-swimmer configurations, show a similar trend. As the swimming speed increases, the drafter experiences an increased wave drag reduction. A higher speed will result in larger wave amplitudes, and the drafter could extract more energy from the waves generated by the leader. At larger transverse distance, e.g. *d_t_* = 2.0 m, *C*_DR_ increases linearly with the swimming speed. At smaller transverse distance, e.g. *d_t_* = 1.5 m, *C*_DR_ increases very slowly at *U* > 1.8 m s^−1^. In two-swimmer case, the wave drag reduction at *U* = 2.0 m s^−1^ is even smaller than that at *U* = 1.9 m s^−1^. This is because the wave drag reduction is not only determined by the wave amplitude, but also by the wavelength. An increased speed will bring a larger wave amplitude, as well as longer waves. At *U* = 1.9 m s^−1^, a better wave-riding configuration is achieved than at *U* = 2.0 m s^−1^. The results also show that when the swimmers are getting closer, the drafter could achieve a higher wave drag reduction. However, at very high swimming speed, the drag reduction becomes less sensitive to the transverse separation.

## 5. Conclusion

Returning to our central questions: (1) what mechanism determines the interaction; (2) which positions experience drag reduction or drag increase; (3) how much can drag be reduced or increased in ‘drafting’? To answer these questions, we established a mathematical and numerical model and calculated the wave drag of a swimmer swimming alone and in formation in open water. Though the answers are highly dependent on the specific swimmer and swimming event, the findings in this study shed light on the importance of the wave interference effects on competitive swimming.

The interaction between human swimmers is determined by the wave interference on the free water surface. The energy-saving position of the drafter is determined by the wave drag reduction. The maximum wave drag reduction is observed when the drafter's fore part is in the wave trough while the aft part is in the wave crest. By taking this wave-riding position, a destructive wave interference phenomenon can be observed, where the waves generated by the swimmers are 180° out of phase. As a result of the wave cancellation effect, the wave drag can be minimized. In a two-swimmer configuration with lateral separation of 2.0 m, the maximum wave drag reduction of the drafter swimming at *U* = 2.0 m s^−1^ is 64%, when the partial wave cancellation effect occurs. In a three-swimmer configuration, a full wave cancellation effect can be observed, where the maximum wave drag reduction achievable is 102%. In this case, the wave drag turns to be a thrust force, pushing the drafter forward. It should be noted that the above conclusions are based on wave drag computations for a simplified model. The effects of fluid viscosity, lane ropes and immersed depth were not taken into account.

The principle finding of this work is that competitive swimmers could experience a strong hydrodynamic interaction when swimming in formation. By swimming in an optimum position behind one/two leading swimmers, the drafter could use the Kelvin waves as a propelling aid to preserve energy, hence improving swimming performance.

## Data accessibility

All the data are shown in the figures of the paper. The original data can be obtained by e-mailing: zhiming.yuan@strath.ac.uk.
